# Drug-coated balloon angioplasty versus balloon angioplasty for treating patients with in-stent restenosis in the femoropopliteal artery: A meta-analysis

**DOI:** 10.1097/MD.0000000000025599

**Published:** 2021-04-23

**Authors:** Shaobo Cao, Tao He, Jinfeng Xie, Haijun Feng, Kui Liu, Bihui Qu, Xiaoling Wu

**Affiliations:** a Department of Vascular Surgery; b Department of Geriatrics, Wuhan Central Hospital, Tongji Medical College, Huazhong University of Science and Technology, Wuhan, China.

**Keywords:** balloon angioplasty, drug coated balloon, femoropopliteal artery, in-stent restenosis, peripheral artery disease

## Abstract

**Background:**

The introduction of endovascular surgery has led to frequent stent use, although in-stent restenosis (ISR) remains a challenging issue. Drug-coated balloon (DCB) and conventional balloon angioplasty (BA) are common endovascular procedures for addressing ISR in the femoropopliteal artery. However, there is controversy regarding which procedure provides the greatest benefit to patients.

**Methods:**

The PubMed, EMBASE, and Cochrane Central Register of Controlled Trials databases were searched for prospective controlled trials that compared DCB and BA for patients with ISR in the femoropopliteal artery. The study has been approved by Ethics Committee of Wuhan Central Hospital.

**Results:**

The meta-analysis included 6 prospective trials with 541 patients. We found that DCB use was associated with significant reductions in binary restenosis at 6 months (relative risk [RR]: 0.45, 95% confidence interval [CI]: 0.33–0.63; *P* < .00001), binary restenosis at 1 year (RR: 0.44, 95% CI: 0.34–0.57; *P* < .00001), target lesion revascularization (TLR) at 6 months (RR: 0.36, 95% CI: 0.20–0.65; *P* = .0006), and TLR at 1 year (RR: 0.38, 95% CI: 0.27–0.54; *P* < .00001). The DCB group also had significantly better clinical improvement (RR: 1.39, 95% CI: 1.13–1.71; *P* = .002), although we did not detect inter-group differences in terms of death, target vessel thrombosis, or ipsilateral amputation. The brand of DCB may a cause of heterogeneity.

**Conclusion:**

Relative to BA, DCB use increases the durability of treatment for ISR in the femoropopliteal artery, based on significant reductions in binary restenosis and TLR at 6–12 months after the procedure. Furthermore, DCB use was associated with better clinical improvement. However, additional randomized controlled trials are needed to validate these findings.

## 1 Introduction

The prevalence of peripheral artery disease (PAD) increases with age, and this disease affects an estimated 20% of people who are >70 years old.^[1]^ Rapid improvements in endovascular instruments and physician experience have led to endovascular therapy being recommended as a first-line option for femoropopliteal artery disease.^[2]^ However, percutaneous intervention still has a limited success rate, especially in patients with stenosis or occlusion of the femoropopliteal arteries, as percutaneous transluminal angioplasty (PTA) is associated with restenosis rates of up to 58% during the first 6 to 12 months.^[3]^ Thus, in-stent restenosis (ISR) has become a challenging issue.^[4]^

Drug-coated balloon (DCB) use has shown promising results in reducing restenosis of coronary stents,^[5,6]^ although there is no standard treatment for ISR in the femoropopliteal artery.^[7]^ Several recent randomized studies have evaluated the safety and efficacy of DCBs among patients with ISR in the femoropopliteal artery,^[8–10]^ although the findings have been inconsistent. Thus, there is no consensus regarding the ideal strategy for treating ISR in the femoropopliteal artery, and we performed a meta-analysis to compare the outcomes of DCB use and balloon angioplasty (BA) in this setting.

## 2 Methods

### 2.1 Search strategy

The PubMed, EMBASE, and the Cochrane Central Register of Controlled Trials databases were searched for English reports of studies that were published before October 2020. The searches were independently performed by two reviewers using the keywords “in-stent restenosis,” “drug coated balloon,” “drug-eluting balloon,” “paclitaxel-eluting balloon,” and “femoral-popliteal arteries.” The reference lists of identified reports and review articles were also examined to identify potentially relevant studies. This study was performed in accordance with the Preferred Reporting Items for Systematic Reviews and Meta-Analysis guidelines.

### 2.2 Study selection

Titles and abstracts were independently screened by two investigators to identify prospective controlled trials that evaluated the outcomes of DCB and BA treatment for patients with ISR in the femoropopliteal artery. Any disagreements regarding a study's eligibility were resolved via re-reading and discussion. The inclusion criteria were:

1. patients with documented ISR in the femoral and/or popliteal arteries and
2. follow-up of ≥6 months.

Studies were not restricted based on the use of any specific type or brand of DCB. The exclusion criteria were:

1. target vessels that were not the femoropopliteal arteries,
2. non-BA and non-DCB treatment, and
3. review articles or duplicate studies.

### 2.3 Data collection and quality assessment

Two reviewers independently extracted and tabulated data from each article regarding baseline demographic characteristics, procedural variables, follow-up time, and primary and secondary endpoints. Data were extracted from the main text and tables of the published reports, as well as from online materials if available. The same authors also evaluated the quality of the included studies using the Cochrane Collaboration's tool for assessing the risk of bias.^[11]^ Any disagreements between the reviewers were resolved via discussion.

### 2.4 Outcome variables

The outcome measures were defined according to previously published reporting standards and were analyzed on an intention-to-treat basis.^[12]^ The primary outcome was defined as the likelihood of target lesion revascularization (TLR). TLR was performed if clinically indicated, and when a target lesion diameter stenosis of 50% was present. Secondary outcomes were defined as binary restenosis, clinical improvement, death, target vessel thrombosis, and ipsilateral amputation. Binary restenosis was defined as a >50% diameter stenosis (by angiography) or a peak systolic velocity ratio 2.5 (by duplex ultrasound). Clinical improvement was defined as at least 1 Rutherford-Becker category observed. Ipsilateral amputation defined as unplanned amputation of the target limb. Death defined as all-cause death.

### 2.5 Statistical analysis

Data were managed and analyzed using Review Manager software (version 5.2; the Cochrane Collaboration) and STATA software (version 12.0; STATA Corporation, College Station). Pooled risk ratios (RR) were calculated for dichotomous variables using the Mantel-Haenszel method and pooled mean differences were calculated for continuous variables. Outcomes were reported with the 95% confidence intervals (CIs) and results were considered statistically significant at *P*-values of <.05.

Heterogeneity was evaluated using the Cochrane Q test.^[13]^ A fixed effects model was used if there was no significant heterogeneity (*P* > .1 and *I*^2^ < 50%), while a random effects model was used if there was significant heterogeneity (*P* < .1 or *I*^2^ > 50%). Sensitivity analyses were performed by excluding each study and re-analyzing the data. Various subgroup analysis was also been carried to address the heterogeneity. Publication bias was evaluated using funnel plots, Begg rank correlation, and the Egger linear regression test,^[14]^ with significant publication bias considered present at *P*-values of <.05.

## 3 Results

### 3.1 Eligible studies

**Figure 1:**
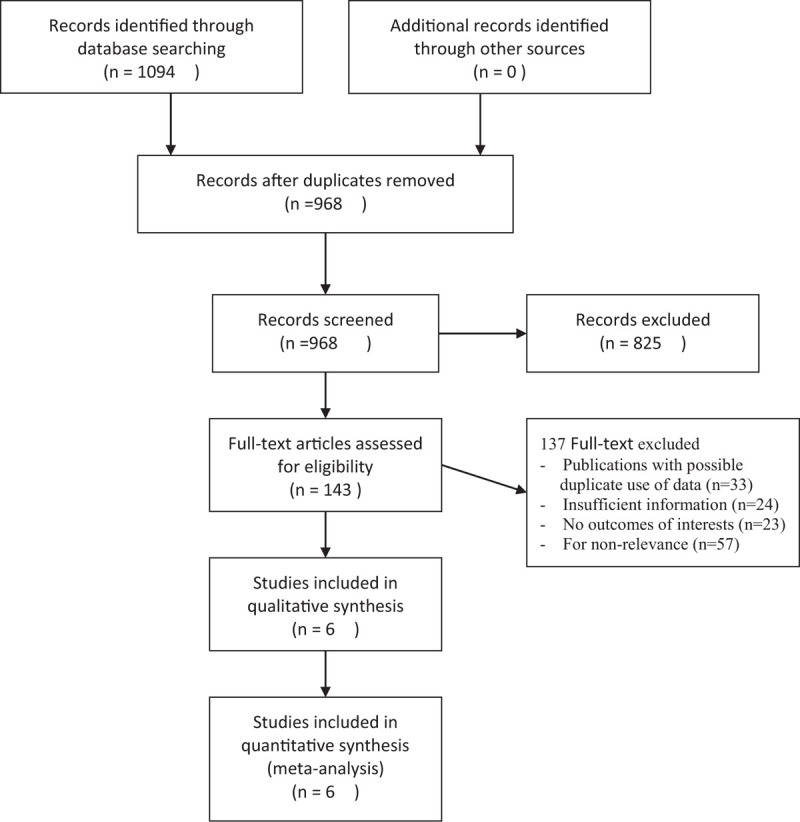
The flow chart of systematic studies search and selection procedure.

The study selection flowchart is shown in Figure 1. The search identified 968 articles, although 825 articles were excluded after a review of the titles and abstracts. Full-text reviews were performed for the remaining 143 articles, and 6 studies with 541 patients ultimately fulfilled the inclusion criteria. Three different brands (Medtronic, FREEWAY, Medrad) of DCBs and 2 different paclitaxel doses (3 and 3.5 ug/mm^2^) were used in the included studies. The main characteristics of the included studies are reported in Table 1. The patients’ demographic characteristics and risk factors are shown in Table 2.

**Table 1:** Characteristics of the included studies.

Trial	Time period	Publication year	Registration no.	Study design	Blind	Inter-ventions	Paclitaxel dose, ug/mm^2^	Primary end point	Second end point	Criteria	Exclusion	Anticoagulation/antiplatelets
ISAR-PEBIS	2010–2013	2017	NCT 01083394	Multicenter RCT	UN	DCB/BA	3.5	The percentage diameter stenosis at 6–8 months	The rate of binary restenosis, the incidence of TLR, major adverse vascular events	Symptomatic ISR > 70% or occlusion of SFA	Acute ischemia, thrombosis, untreated ipsilateral iliac artery stenosis >70%, severe renal insufficiency, life expectancy <1 year, contraindication to study medications	Aspirin 100 mg/day indefinitely and clopidogrel 75 mg/day for at least 6 months
FAIR	2010–2012	2015	NCT 01305070	Multicenter RCT	Non-blinded	DCB/BA	3.5	Recurrent binary recurrent restenosis at 6 months	Binary recurrent restenosis at 12 months, freedom from TLR, ABI, clinical improvement, major adverse vascular events	A SFA ISR up to 20 cm, stenosis >70%; one infrapopliteal for distal runoff; Rutherford category 2–4.	An untreated ipsilateral iliac artery stenosis; ongoing dialysis treatment; treatment with oral anticoagulants	Aspirin 100 mg/d indefinitely plus clopidogrel 75 mg/d for at least 6 months
PACUBA	2010–2012	2016	NCT 01247402	Multicenter RCT	Single blind	DCB/BA	3	Recurrent binary recurrent restenosis	Technical success, complication rate, clinical success, change in ABI, freedom from TLR at 6 and 12 months	Age > 50 years, symptomatic PAD, ISR > 50% in the SFA and P1 segment of the popliteal artery, at least 1 patent tibial vessel with distal runoff, Rutherford category 2–3	Inability to write informed consent; contraindication to study medications; and creatinine > 2.5 mg/dL	Aspirin 100 mg/day indefinitely and clopidogrel 75 mg/day for 3 months
DEBATE-SFA	2010–2011	2013	NCT 01556542	Single center RCT	Non-blinded	DCB + BMS/BA + BMS	3	Recurrent binary restenosis at 12 months	The incidence of TLR, major amputation at 12 months	de novo stenosis 50%, occlusion of at least 40 mm in the SFA; a clear segment between the lesion in the SFA and common femoral artery and between the popliteal and tibioperoneal trunk; at least 1 patent tibial vessel with distal runoff	Life expectancy <1 year; any contraindication to study medications; need for major amputation at the time of enrollment. Failure to recanalize intended below-the-knee arteries at risk of major amputation	Aspirin 100 mg/day plus clopidogrel 75 mg/day 1 month and 3 months
DEBATE-ISR	2010–2011	2014	NCT 01558531	Prospective	UN	DCB/BA	3	Recurrent binary restenosis at 12 months	Freedom from TLR. clinical improvement, major adverse events	Diabetic patients with femoropopliteal ISR	Paclitaxel allergy; contraindication to combined antiplatelet treatment; life expectancy < 1 year	Aspirin 100 mg/d plus clopidogrel 75 mg/d 6 months
COPA CABANA	2011–2013	2020	NCT 01594684	Multicenter RCT	Non-blinded	DCB/BA	3	LLL at 6 months	The incidence of TLR	ISR ≥70% or in-stent occlusion 3–27 cm long in the SFA and/or popliteal artery occurring >3 months after stent implantation; Rutherford category 2–5; at least 1 patent runoff vessel	Subintimal approach to the ISR lesion; presence of stent fracture; planned major amputation; aneurysm in the target vessel; Severe abnormalities in platelet and leukocyte counts contraindication to study medications	Clopidogrel 75 mg/d continued for 4 weeks with lifelong aspirin 100 mg/d

**Table 2:** Demographics and risk factors of patients.

Trial	Interventions	Brand of device	Patients	Age	Male	Diabetes (n)	Hypertension (n)	Smoking (n)	CAD (n)	Renal failure (n)	ABI	RVD	Rutherford class > 4 (n)	Follow up (month)
ISAR-PEBIS	DCB	In.Pact Admiral (Medtronic)	36	70 ± 10	24/36	12	33	21	17	UN	0.6 ± 0.3	4.8 ± 1.3	1	24
	BA	Pacific Xtreme (Medtronic)	34	68 ± 10	24/34	12	30	24	16	UN	0.7 ± 0.2	4.8 ± 1.2	1	24
FAIR	DCB	In.Pact^TM^ Admiral (Medtronic)	62	69 ± 8	33	28	52	18	26	8	0.63 ± 0.27	5.1 ± 0.9	3	12
	BA	Admiral Xtreme (Medtronic)	57	67 ± 9	49	17	53	20	22	10	0.64 ± 0.25	5.4 ± 0.5	6	12
PACUBA	DCB	FREEWAY 0.035 DCB (Eurocor)	35	68.1 ± 9.2	20	17	26	17	12	6	0.65 ± 0.16	5.7 ± 0.1	0	12
	BA	unspecified	39	68.3 ± 0.4	23	13	27	18	14	6	0.65 ± 0.116	5.4 ± 0.9	0	12
DEBATE-SFA	DCB + BMS	In.Pact Admiral Invatec (Medtronic)	53	74 ± 9	40	41	47	25	21	5	0.33 ± 0.22	5.01 ± 0.5	42	12
	BA + BMS	unspecified	51	76 ± 8	32	36	45	28	18	3	0.31 ± 0.18	5.12 ± 0.5	35	12
DEBATE-ISR	DCB	In.Pact Admiral (Medtronic)	44	32	32	44	39	14	9	UN	0.32 ± 0.11	4.9 ± 0.4	33	36
	BA	unspecified	42	23	23	42	38	11	12	UN	0.36 ± 0.9	5.0 ± 0.5	28	36
COPA CABANA	DCB	Cotavance DCB (Medrad)	47	68.3 ± 9.6	26	20	38	14	10	UN	0.72 ± 0.23	5.2 ± 0.6	3	22
	BA	unspecified	41	67.6 ± 10.2	26	19	30	15	10	UN	0.65 ± 0.25	5.1 ± 0.8	5	21.9

### 3.2 Risk of bias

**Figure 2:**
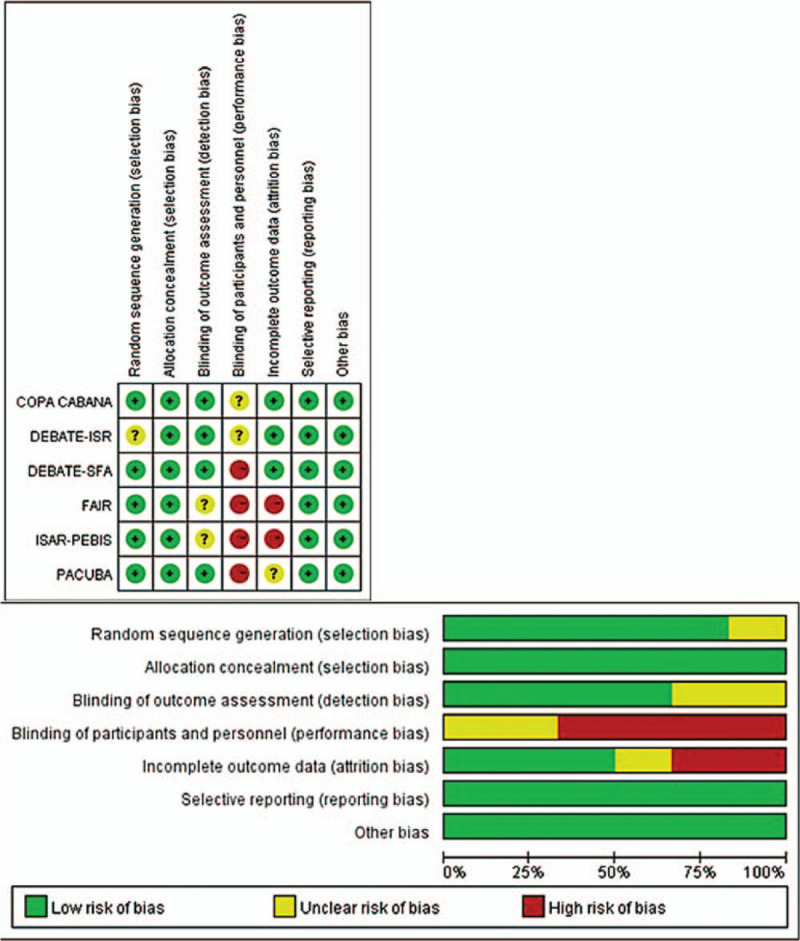
Risk of bias assessment.

The findings regarding risk of bias are summarized in Figure 2. There were low risks of selection bias and reporting bias for all trials, although 4 trials had high risks of performance bias, which was the most prominent quality issue. Two studies with >2 risk factors for bias were considered low-quality, although the other studies were considered high-quality and the results were considered applicable.

### 3.3 Clinical outcomes

#### 3.3.1 TLR

**Figure 3:**
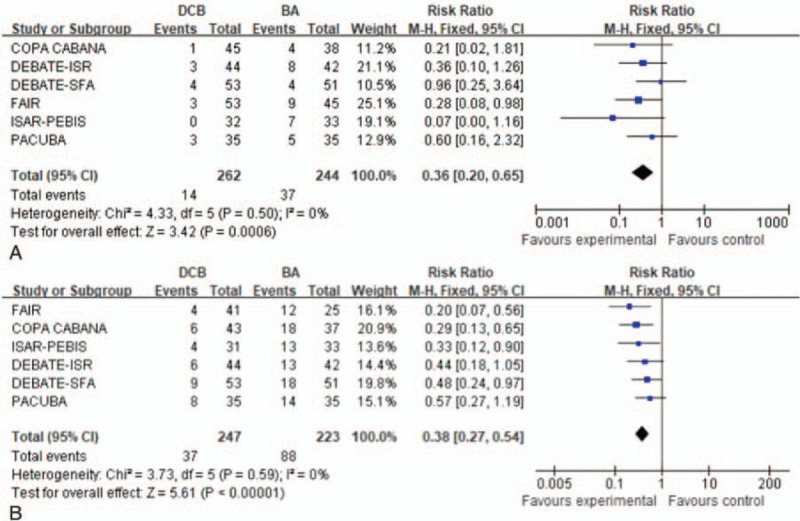
Forest plots of risk ratio of target lesion revascularization at 6 months (A) and 12 months (B).

Six trials^[15–20]^ with 541 patients provided data regarding TLR at 6 months. A fixed effects model was used based on the absence of significant heterogeneity (*P* = .5, *I*^2^ = 0%). The use of DCBs was associated with a significantly reduced risk of TLR at 6 months (RR: 0.36, 95% CI: 0.20–0.65; *P* = .0006) (Fig. 3A).

Six trials^[15–20]^ also provided data regarding TLR at 12 months. A fixed effects model was used based on the absence of significant heterogeneity (*P* = 0.59, *I*^2^ = 0%). The use of DCBs was associated with a significantly reduced risk of TLR at 12 months (RR: 0.38, 95% CI: 0.27–0.54; *P* < .00001) (Fig. 3B).

##### 3.3.1.1 Binary restenosis

**Figure 4:**
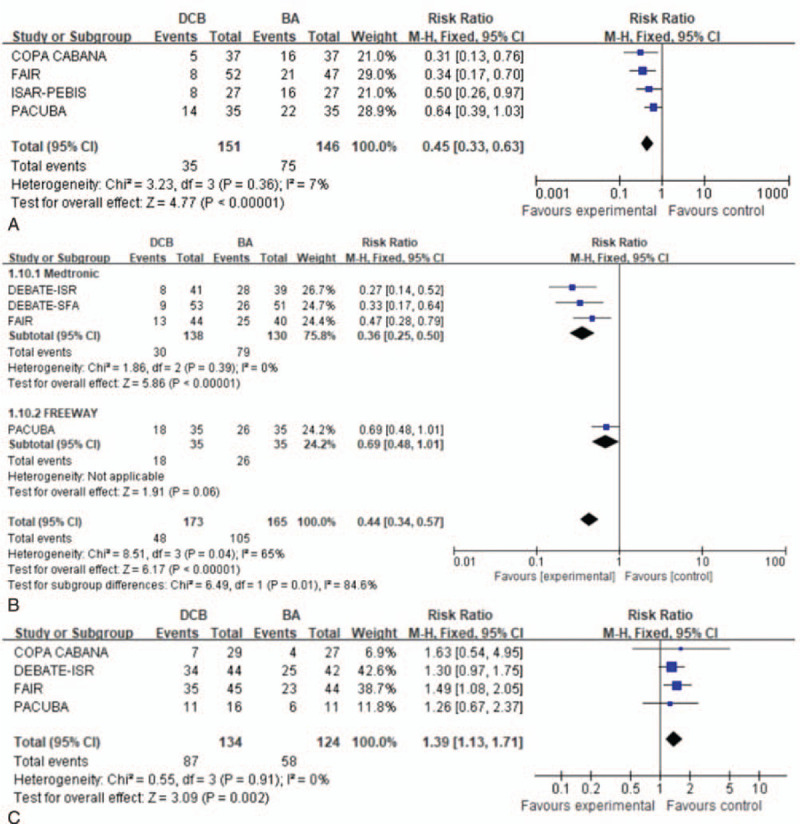
Forest plots of risk ratio of binary restenosis at 6 months (A). Forest plots of pooled estimates of binary restenosis including Medtronic and FREEWAY subgroup analysis at 12 months (B). Forest plots of risk ratio of clinical improvement at 12 months (C).

Four trials^[15–17,20]^ evaluated the risk of binary restenosis at 6 months. A fixed effects model was used based on the absence of significant heterogeneity (*P* = .36, *I*^2^ = 7%). The use of DCBs was associated with a significantly reduced risk of binary restenosis at 6 months (RR: 0.36, 95% CI: 0.33–0.63; *P* < .00001) (Fig. 4A).

Four trials^[16–19]^ evaluated the risk of binary restenosis at 12 months. A moderate heterogeneity was found in random effects forest plots (*P* = .04, *I*^2^ = 65%). Sensitivity analyses found that the PACUBA research^[17]^ which using the DCB of FREEWAY was the cause of heterogeneity. Subgroup analysis of different brand (Medtronic, FREEWAY) was showed in Figure 4B. There was zero statistical heterogeneity within the brand subgroups (*P* = .39, *I*^2^ = 0). The use of Medtronic DCBs subgroup was associated with a significantly reduced risk of binary restenosis at 12 months (RR: 0.36, 95% CI: 0.25–0.50; *P* < .00001). The use of FREEWAY DCB subgroup reduce the risk of binary restenosis at 12 months, but no statistically significance found (RR: 0.69, 95% CI: 0.48–1.01; *P* = .06) (Fig. 4B). There was high-quality evidence that DCBs significantly reduce risk of binary restenosis at 12 months in the overall pool of trials (RR: 0.44, 95% CI: 0.34–0.57; *P* < .00001).

##### 3.3.1.2 Clinical improvement

Four trials^[16,17,19,20]^ evaluated clinical improvement at 12 months. A fixed effects model was used based on the absence of significant heterogeneity (*P* = .91, *I*^2^ = 0%). The DCB group had significantly better clinical improvement (RR: 1.39, 95% CI: 1.13–1.71; *P* = .002) (Fig. 4C).

### 3.4 Major adverse events

#### 3.4.1 Death

**Figure 5:**
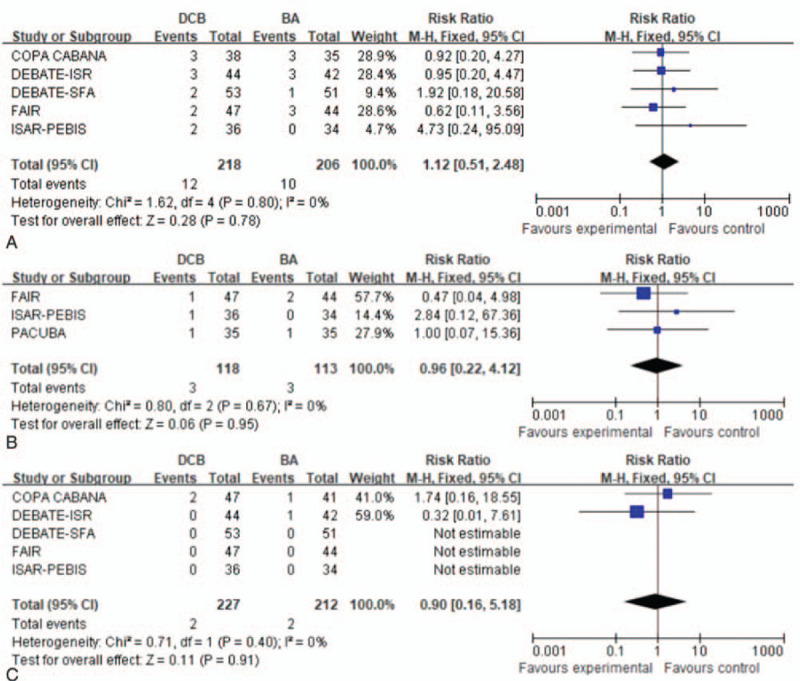
Forest plots of risk ratio of mortality at 12 months (A). Forest plots of risk ratio of target vessel thrombosis at 12 months (B). Forest plots of risk ratio of ipsilateral amputation rate at 12 months (C).

Five trials^[15,16,18–20]^ evaluated the mortality rate at 12 months. A fixed effects model was used based on the absence of significant heterogeneity (*P* = .8, *I*^2^ = 0%). There was no significant difference in mortality rate between the DCB and BA treatment groups (RR: 1.12, 95% CI: 0.51–2.48; *P* = .78) (Fig. 5A).

#### 3.4.2 Target vessel thrombosis

Three trials^[15–17]^ evaluated target vessel thrombosis at 12 months. A fixed effects model was used based on the absence of significant heterogeneity (*P* = .67, *I*^2^ = 0%). There was no significant difference in target vessel thrombosis between the DCB and BA treatment groups (RR: 0.96, 95% CI: 0.22–4.12; *P* = .95) (Fig. 5B).

#### 3.4.3 Amputation rate

Five trials^[15,16,18–20]^ evaluated the ipsilateral amputation rate at 12 months, which revealed ipsilateral amputation for 4 patients within 12 months (2 patients in the BA group and 2 patients in the DCB group). There was no significant difference in the ipsilateral amputation rate (RR: 0.90, 95% CI: 0.16–5.18; *P* = .91) (Fig. 5C).

### 3.5 Publications bias and heterogeneity analysis

**Figure 6:**
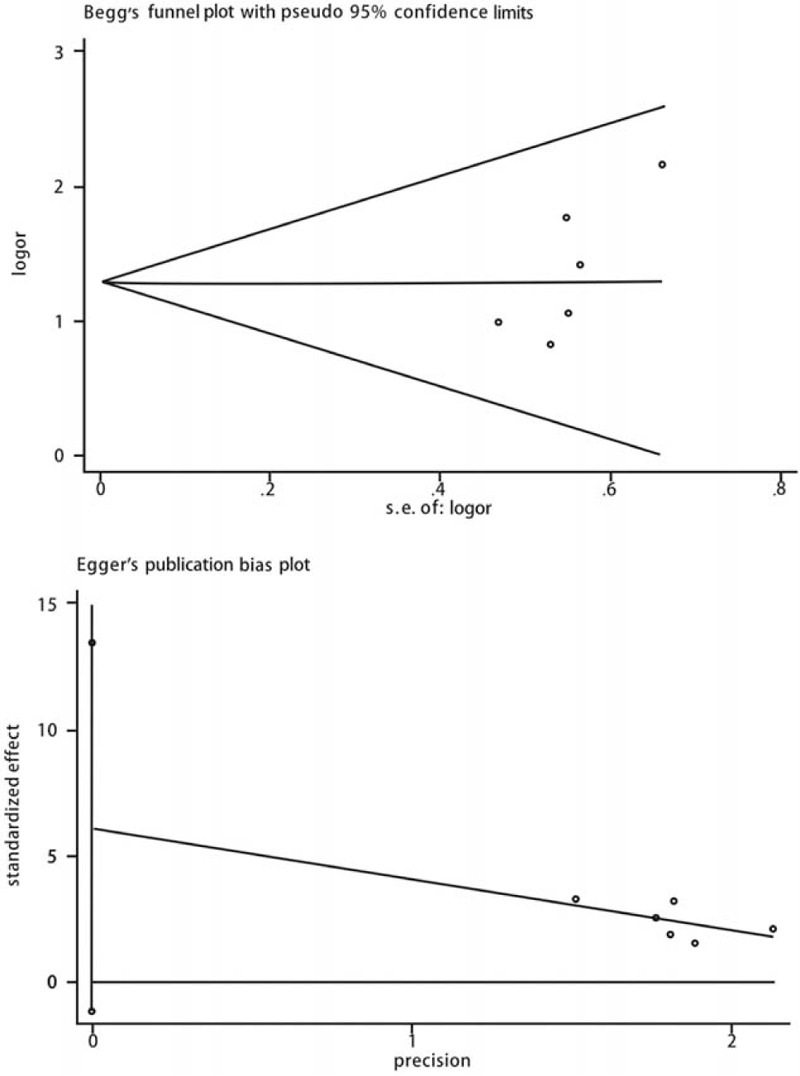
Begg's and Egger's publication bias plots of the included studies.

A visual inspection of the funnel plot did not reveal any clear asymmetry. Similarly, no significant publication bias was detected using the Egger and Begg tests (*P* = .22) (Fig. 6). Sensitivity analyses, which involved omitting one study at a time from the meta-analysis, failed to indicate that the results were influenced by a particular study. Subgroup analysis showed that study designs (RCT, and prospective study) and paclitaxel dose (3 and 3.5 ug/mm^2^) were not the cause of heterogeneity. Brand (Medtronic, FREEWAY) of the DCB may be a heterogeneity cause in the binary restenosis result.

## 4 Discussion

The main disadvantage of PTA and stenting is that high rates of ISR can significantly affect the clinical outcomes of femoropopliteal artery stenting.^[21–23]^ Furthermore, the 1-year rates of ISR range from 18% to 37%.^[24]^ Thus, the increased use of endovascular therapy makes ISR and its treatment a challenging issue.^[23]^

The most recent meta-analysis included 3 trials with 263 patients, and revealed that DCB use provided advantages (vs uncoated BA) for treating ISR in the superficial femoral artery based on rates of TLR, binary restenosis, and clinical improvement within 2 years after the procedure. However, the level of evidence was considered low, based on the small number of studies and potential risks of bias.^[25]^ In addition, the evidence was considered insufficient in practice to confirm the superiority of DCB over BA for treating ISR, given the high cost of DCBs.^[25]^ Our review included larger numbers of trials and patients, and the results revealed that the DCB treatment group had significantly better outcomes in terms of the 6- to 12-month rates of TLR, binary restenosis, and clinical improvement. We also evaluated the adverse events for each treatment, which revealed similar amputation and mortality rates in the DCB and BA groups. Unfortunately, few studies have provided data regarding the costs during the follow-up period, although previous meta-analyses also support our conclusion to some extent.^[26–28]^ Katsanos et al^[26]^ reported that DCBs provided a >50% reduction in the rates of restenosis (including ISR) and TLR in the femoropopliteal artery, and suggested that standard paclitaxel dose (3.0 and 3.5 ug/mm^2^) DCBs were more effective compared with low paclitaxel dose (2.0 ug/mm^2^) in reduce both restenosis and TLR. In our meta-analysis, low paclitaxel dose subgroup (3.0 ug/mm^2^) and high paclitaxel dose subgroup (3.5 ug/mm^2^) were both superior to BA in restenosis and TLR. The differences between the two subgroups were hard to analysis, because of the small number of the included studies. Anantha-Narayanan et al also reported that the TLR rates (including for ISR) in the femoropopliteal artery were 45% lower in the DCB group than in the BA group. Tepe et al^[20]^ reported a recent randomized controlled trial, which has not been included in previous meta-analyses, which revealed that DCB use was associated with significantly less late lumen loss and fewer TLR procedures up to 24 months after treatment. To the best of our knowledge, ours is the most comprehensive meta-analysis to compare the outcomes of DCB and BA treatment for ISR in the femoropopliteal artery.

There are limited data regarding DCB use for PAD, although the existing evidence seems to indicate that DCB use is associated with a significant benefit.^[4,29,30]^ The first cohort study revealed a 92.1% primary patency rate at 12 months after treatment using DCBs,^[9]^ while a more recent study of 53 patients with ISR in the femoropopliteal artery revealed a primary patency rate of 83.7% ± 5.0% and ∼90% freedom from TLR after 1 year.^[31]^ The results of these 2 trials suggest that DCBs are a promising treatment option for patients with ISR in the femoropopliteal artery, which agrees with our findings, although it is important to note that both studies did not have a control group.

Various strategies have been used to treat ISR in the femoropopliteal artery. One randomized study revealed that, relative to DCB alone, a combination of laser debulking and DCB provided significantly better patency rates at 6 months (91.7% vs 58.3%; *P* = .01) and at 12 months (66.7% vs 37.5%; *P* = .01).^[32]^ Bosiers et al compared the ISR rates after treatment using PTA and Viabahn ePTFE-covered stents, which revealed that the Viabahn stents provided better 1-year rates of primary patency (74.8% vs 28%; *P* < .001) and freedom from TLR (80% vs 42%; *P* < .001).^[33]^ Silverhawk atherectomy is not superior to PTA, as it was associated with increased reoccurrence of intimal media hyperplasia.^[34]^ Drug-eluting stents are a questionable treatment for ISR, as the scaffolding is not needed to manage the migration, proliferation, and collagen synthesis of smooth muscle cells.^[31]^ Cutting balloons are also not superior to conventional PTA.^[35]^

Our meta-analysis has some important limitations. First, the pooled analysis was based on study-level data, which could be confounded by inaccurate or incomplete data reporting. Second, the analysis only included a small number of studies with relatively short follow-up periods, which might be inadequate for detecting late adverse events, such as amputation, death, and very late thrombosis. Third, we only considered reports that were published in English, which is a potential source of bias.

## 5 Conclusion

In conclusion, our results indicate that DCB use was superior to BA for treating patients with ISR in the femoropopliteal artery, based on significantly better 6- to 12-month rates of binary restenosis, TLR, and clinical improvement. Furthermore, the rates of amputation and mortality were similar between the DCB and BA treatment groups. However, additional randomized controlled trials are needed to validate these findings.

## Author Contributions

**Conceptualization:** Bihui Qu.

**Data curation:** Shaobo Cao, Xiaoling Wu.

**Formal analysis:** Shaobo Cao, Jinfeng Xie, Haijun Feng.

**Methodology:** Shaobo Cao, Xiaoling Wu.

**Software:** Xiaoling Wu, Jinfeng Xie, Haijun Feng.

**Supervision:** Kui Liu, Bihui Qu, Tao He.

**Validation:** Kui Liu, Bihui Qu.

**Writing – original draft:** Shaobo Cao, Xiaoling Wu.

**Writing – review & editing:** Shaobo Cao, Xiaoling Wu, Tao He.

## References

[1] Steiner S Schmidt A Bausback Y. Single-center experience with Lutonix drug-coated balloons in infrapopliteal arteries. J Endovasc Ther 2016;23:417–23. 27099285 10.1177/1526602816645080

[2] Gerhard-Herman MD Gornik HL Barrett C. 2016 AHA/ACC guideline on the management of patients with lower extremity peripheral artery disease: executive summary: a report of the American College of Cardiology/American Heart Association Task Force on Clinical Practice Guidelines. Circulation 2017;135:e686–725. 27840332 10.1161/CIR.0000000000000470 PMC5479414

[3] Herten M Torsello GB Schonefeld E. Critical appraisal of paclitaxel balloon angioplasty for femoral-popliteal arterial disease. Vasc Health Risk Manag 2016;12:341–56. 27621646 10.2147/VHRM.S81122 PMC5010165

[4] Andrassy M Celik S Andrassy J. The role of drug-coated balloons in in-stent restenosis. J Cardiovasc Surg (Torino) 2017;58:501–7. 10.23736/S0021-9509.17.09963-3 28358184

[5] Unverdorben M Vallbracht C Cremers B. Paclitaxel-coated balloon catheter versus paclitaxel-coated stent for the treatment of coronary in-stent restenosis: the three-year results of the PEPCAD II ISR study. EuroIntervention 2015;11:926–34. 25169589 10.4244/EIJY14M08_12

[6] Scheller B Hehrlein C Bocksch W. Treatment of coronary in-stent restenosis with a paclitaxel-coated balloon catheter. N Engl J Med 2006;355:2113–24. 17101615 10.1056/NEJMoa061254

[7] Tosaka A Soga Y Iida O. Classification and clinical impact of restenosis after femoropopliteal stenting. J Am Coll Cardiol 2012;59:16–23. 22192663 10.1016/j.jacc.2011.09.036

[8] Scheinert D Schulte KL Zeller T. Paclitaxel-releasing balloon in femoropopliteal lesions using a BTHC excipient: twelve-month results from the BIOLUX P-I randomized trial. J Endovasc Ther 2015;22:14–21. 25775674 10.1177/1526602814564383

[9] Stabile E Virga V Salemme L. Drug-eluting balloon for treatment of superficial femoral artery in-stent restenosis. J Am Coll Cardiol 2012;60:1739–42. 23040582 10.1016/j.jacc.2012.07.033

[10] Werk M Langner S Reinkensmeier B. Inhibition of restenosis in femoropopliteal arteries: paclitaxel-coated versus uncoated balloon: femoral paclitaxel randomized pilot trial. Circulation 2008;118:1358–65. 18779447 10.1161/CIRCULATIONAHA.107.735985

[11] Higgins JP Altman DG Gotzsche PC. The Cochrane Collaboration's tool for assessing risk of bias in randomised trials. BMJ 2011;343:d5928. 22008217 10.1136/bmj.d5928 PMC3196245

[12] Sacks D Marinelli DL Martin LG. Society of Interventional Radiology Technology Assessment C. Reporting standards for clinical evaluation of new peripheral arterial revascularization devices. J Vasc Interv Radiol 2003;14:S395–404. 14514855 10.1097/01.rvi.0000094613.61428.a9

[13] Bowden J Tierney JF Copas AJ. Quantifying, displaying and accounting for heterogeneity in the meta-analysis of RCTs using standard and generalised Q statistics. BMC Med Res Methodol 2011;11:41. 21473747 10.1186/1471-2288-11-41 PMC3102034

[14] Egger M Davey Smith G. Bias in meta-analysis detected by a simple, graphical test. BMJ 1997;315:629–34. 9310563 10.1136/bmj.315.7109.629 PMC2127453

[15] Ott I Cassese S Groha P. ISAR-PEBIS (Paclitaxel-Eluting Balloon Versus Conventional Balloon Angioplasty for In-Stent Restenosis of Superficial Femoral Artery): a randomized trial. J Am Heart Assoc 2017;25:e006321. 10.1161/JAHA.117.006321 PMC5586321 28743787

[16] Krankenberg H Tübler T Ingwersen M. Drug-coated balloon versus standard balloon for superficial femoral artery in-stent restenosis: the randomized Femoral Artery In-Stent Restenosis (FAIR) trial. Circulation 2015;132:2230–6. 26446728 10.1161/CIRCULATIONAHA.115.017364

[17] Kinstner CM Lammer J Willfort-Ehringer A. Paclitaxel-eluting balloon versus standard balloon angioplasty in in-stent restenosis of the superficial femoral and proximal popliteal artery: 1-year results of the PACUBA trial. JACC Cardiovasc Interv 2016;9:1386–92. 27388828 10.1016/j.jcin.2016.04.012

[18] Liistro F Grotti S Porto I. Drug-eluting balloon in peripheral intervention for the superficial femoral artery: the DEBATE-SFA randomized trial (drug eluting balloon in peripheral intervention for the superficial femoral artery). JACC Cardiovasc Interv 2013;6:1295–302. 24239203 10.1016/j.jcin.2013.07.010

[19] Liistro F Angioli P Porto I. Paclitaxel-eluting balloon vs. standard angioplasty to reduce recurrent restenosis in diabetic patients with in-stent restenosis of the superficial femoral and proximal popliteal arteries: the DEBATE-ISR study. J Endovasc Ther 2014;21:01–8. 10.1583/13-4420R.1 24502477

[20] Tepe G Schroeder H Albrecht T. Paclitaxel-coated balloon vs uncoated balloon angioplasty for treatment of in-stent restenosis in the superficial femoral and popliteal arteries: the COPA CABANA trial. J Endovasc Ther 2020;27:276–86. 32096451 10.1177/1526602820907917

[21] Laird JR Schneider PA Tepe G. Durability of treatment effect using a drug-coated balloon for femoropopliteal lesions: 24-month results of IN.PACT SFA. J Am Coll Cardiol 2015;66:2329–38. 26476467 10.1016/j.jacc.2015.09.063

[22] van den Berg JC. In-stent restenosis management: the best is yet to come. J Cardiovasc Surg (Torino) 2017;58:508–17. 10.23736/S0021-9509.17.09953-0 28322039

[23] Kondapalli A Danek BA Khalili H. Drug-coated balloons: current outcomes and future directions. Interv Cardiol Clin 2017;6:217–25. 28257769 10.1016/j.iccl.2016.12.005

[24] Sobieszczyk P. In-stent restenosis after femoropopliteal interventions with drug-eluting stents: same but different? JACC Cardiovasc Interv 2016;9:835–7. 27101909 10.1016/j.jcin.2016.02.015

[25] Kayssi A Al-Jundi W Papia G. Drug-eluting balloon angioplasty versus uncoated balloon angioplasty for the treatment of in-stent restenosis of the femoropopliteal arteries. Cochrane Database Syst Rev 2019;26:CD012510. 10.1002/14651858.CD012510.pub2 PMC6353053 30684445

[26] Katsanos K Spiliopoulos S Paraskevopoulos I. Systematic review and meta-analysis of randomized controlled trials of paclitaxel-coated balloon angioplasty in the femoropopliteal arteries: role of paclitaxel dose and bioavailability. J Endovasc Ther 2016;23:356–70. 26823485 10.1177/1526602815626557

[27] Wu R Li Z Wang M. Paclitaxel-coated versus uncoated balloon angioplasty for femoropopliteal artery in-stent restenosis. Int J Surg 2017;42:72–82. 28461145 10.1016/j.ijsu.2017.04.057

[28] Anantha-Narayanan M Shah SM Jelani QU. Drug-coated balloon versus plain old balloon angioplasty in femoropopliteal disease: an updated meta-analysis of randomized controlled trials. Catheter Cardiovasc Interv 2019;94:139–48. 30838719 10.1002/ccd.28176

[29] Taniyama Y Jang S-J Hsieh C-A. Feasibility and clinical outcomes of peripheral drug-coated balloon in high-risk patients with femoropopliteal disease. PLoS One 2015;10:e0143658. 26599128 10.1371/journal.pone.0143658 PMC4658025

[30] Christof K Thomas L René A. Benefit and risk from paclitaxel-coated balloon angioplasty for the treatment of femoropopliteal artery disease: a systematic review and meta-analysis of randomised controlled trials. EClinicalMedicine 2019;16:42–50. 31832619 10.1016/j.eclinm.2019.09.004 PMC6890981

[31] Bague N Julia P Sauguet A. Femoropopliteal in-stent restenosis repair: midterm outcomes after paclitaxel eluting balloon use (PLAISIR Trial). Eur J Vasc Endovasc Surg 2017;53:106–13. 27890526 10.1016/j.ejvs.2016.10.002

[32] Gandini R Del Giudice C Merolla S. Treatment of chronic SFA in-stent occlusion with combined laser atherectomy and drug-eluting balloon angioplasty in patients with critical limb ischemia: a single-center, prospective, randomized study. J Endovasc Ther 2013;20:805–14. 24325697 10.1583/13-4308MR.1

[33] Bosiers M Deloose K Callaert J. Superiority of stent-grafts for in-stent restenosis in the superficial femoral artery: twelve-month results from a multicenter randomized trial. J Endovasc Ther 2015;22:01–10. 10.1177/1526602814564385 25775672

[34] Brodmann M Rief P Froehlich H. Neointimal hyperplasia after Silverhawk atherectomy versus percutaneous transluminal angioplasty (PTA) in femoropopliteal stent reobstructions: a controlled, randomized pilot trial. CardioVasc Intervent Radiol 2012;36:69–74. 23007223 10.1007/s00270-012-0479-9

[35] Dick P1 Sabeti S Mlekusch W. Conventional balloon angioplasty versus peripheral cutting balloon angioplasty fortreatment of femoropopliteal artery in-stent restenosis: initial experience. Radiology 2008;248:297–302. 18566179 10.1148/radiol.2481071159

